# Rapid cholera outbreak control following catastrophic landslides and floods: A case study of Bududa district, Uganda

**DOI:** 10.4314/ahs.v23i4.23

**Published:** 2023-12

**Authors:** Godfrey Bwire, Imelda Tumuhairwe, Leocadia Kwagonza, Milton Makoba Wetaka, Anne Nakinsige, Emmanuel Samuel Arinitwe, Julian Kemirembe, Allan Muruta, Charles Mugero, Christine K Nalwadda, Samuel I Okware

**Affiliations:** 1 Ministry of Health, Department of Integrated Epidemiology, Surveillance and Public Health Emergencies, Kampala, Uganda; 2 Department of Health, Bududa District Local Government, Uganda; 3 Ministry of Health, Department of Community Health, Kampala, Uganda; 4 World Health Organization, Pretoria, South Africa; 5 Makerere University, College of Health Sciences School of Public Health, Kampala, Uganda; 6 Uganda National Health Research Organization, Kampala, Uganda

**Keywords:** Cholera, outbreak, vaccination, Africa, Uganda, disaster, landslides, epidemic, floods

## Abstract

**Background:**

In June 2019, landslides and floods in Bududa district, eastern Uganda, claimed lives and led to a cholera outbreak. The affected communities had inadequate access to clean water and sanitation.

**Objective:**

To share the experience of controlling a cholera outbreak in Bududa district, after landslides and floods.

**Methods:**

A descriptive cross-sectional study was carried out in which outbreak investigation reports, weekly epidemiological data and disaster response reports were reviewed.

**Results:**

On 4 – 5th June 2019, heavy rainfall resulted in four landslides which caused six fatalities, 27 injuries, floods and displaced 480 persons. Two weeks later, a cholera outbreak was confirmed in Bududa district. The Ministry of Health (MoH) rapidly deployed oral cholera vaccine (OCV) from local reserves and mass vaccinated 93% of the target population in 22 affected parishes. The outbreak was controlled in 10 weeks with 67 cholera cases and 1 death reported. However, WaSH conditions remained poor, with only, 24.2 % (879/3,628) of the households with washable latrines, 26.8% (1,023/3,818) had hand-washing facilities with soap and 33.6% (1617/4807) used unsafe water.

**Conclusion:**

The OCV stockpile by the MoH helped Uganda to control cholera promptly in Bududa district. High-risk countries should keep OCV reserves for emergencies.

## Introduction

Landslides are a major natural disaster that can trigger cholera outbreaks due to poor water, sanitation and hygiene (WaSH) conditions that are associated with them 1. Cholera, an infection due a bacteria *Vibrio cholerae* is an ancient intestinal disease that is a major public health problem in many developing countries 2. In 2021, 35 countries reported a total of 223,370 cholera cases and 4,159 deaths, to the World Health Organization (WHO) 3. Cholera is preventable; however, epidemics are most frequent in developing countries in sub-Saharan Africa, Asia and Central America 4. Big cholera outbreaks have occurred after natural disasters like in Haiti following the earthquake of 2010 5,6, Mozambique after the floods of 2008 7 and during some manmade disasters like in Yemen 8,9, and South Sudan 10 during the civil wars. Whatever the circumstance that result in cholera, the method of prevention of cholera is by provision of adequate WaSH 11. When WaSH is inadequate and possibility of quick improvement is low, the WHO recommends mass vaccination with oral cholera vaccination (OCV) to prevent cholera outbreak 12–15. The usual source of the OCV is the WHO stockpile that is controlled by a team of experts based in Geneva, Switzerland 16. The countries at risk of cholera that need OCV are required to apply for it from the stockpile in Geneva. The application process for the OCV involve sharing of some data on cholera with the Global Task Force for Cholera Control (GTFCC), WHO, Geneva. Sometimes, due to disruption social services and infrastructure resulting from the disaster, the affected communities/countries may not be in position to access emails and timely respond with required information. Hence, the process of application for OCV may take several weeks and even months 16,17. Often, the vaccines arrive in the cholera affected areas when the outbreak has spread so much or is on declining trend 17,18.

Though cholera is rare in developed countries, it is a major cause of morbidity in East Africa and Uganda in particular 19,20. In Uganda, cholera outbreaks often occur in the districts located along the major lakes 21,22, communities found in areas around the international borders 23,24, those hosting the refugees 17,25 or where there is inadequate safe water 26. Several districts in eastern Uganda are prone to recurrent landslides often following heavy rains which result in loss of life and property, ill health and disruption of water supply and sanitation 27. Bududa district is one such district that is prone to landslides and associated consequences 27,28. Cholera outbreaks and related diarrheal diseases are a major consequence of landslides in Bududa and surrounding districts such as Mbale and Bulambuli 29-31. Though landslides occur frequently in Bududa district 27,32,33 and are followed sometimes by cholera outbreaks, few studies have documented the occurrence of these cholera outbreaks and the response instituted in such setting to control them.

In June 2019, Bududa district experienced two days of continuous torrential rainfall which triggered catastrophic landslides in several parishes in the district. Apart from causing many fatalities, the heavy rains and the landslides destroyed water sources, sanitary facilities, houses, roads, domestic and public property, disrupted service delivery and displaced many people. This scenario created suitable condition for outbreaks of infectious diseases. Two weeks after the landslides, cholera was reported. Consequently, the communities in Bududa district experienced multiple natural disasters simultaneously. The objective of this study was to document the lessons learnt during response to a cholera outbreak in Bududa district that was triggered by the catastrophic landslides and floods.

## Methods

### Study design

A descriptive cross-sectional study was carried out to document lessons learned during response to cholera outbreak in Bududa district for the period June – August 2019. The authors extracted and reviewed Bududa district epidemiological data (a cholera line list and Bududa district cholera daily situational reports) and the landslide disaster response reports for this period.

### Study area

**Figure 1:**
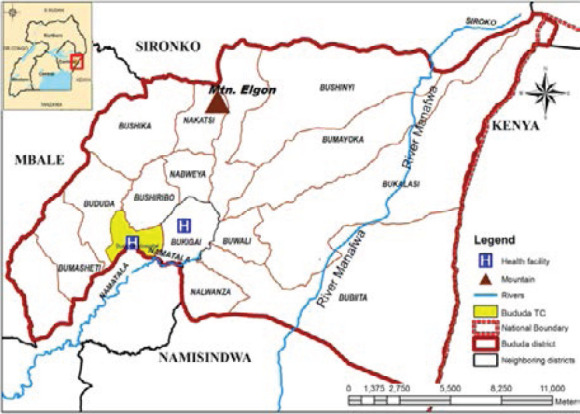
Map of Bududa district showing the sub counties and the major rivers

The study was conducted in Bududa district, eastern Uganda. Bududa district is located on the slopes of Mount (Mt.) Elgon. Mt. Elgon is a massive 80-kilometer diameter solitary volcanic mountain on the eastern border of Uganda and western Kenya. Wagagai peak, 01°07′06″N 34°31′35″E, 4,321 metres above sea level and found in Uganda is the highest point of Mt. Elgon 34. Bududa district has total surface area of 241,551square kilometers 35 with projected population from 2014 census of 259,800 people in 2019 36,37. In terms of administrative structure, Bududa district consisted of two counties, 18 sub-counties, 96 Parishes and 955 villages 36. Bududa Town Council is the biggest town and the district headquarters. Bududa district has mountain vegetation and terrain. The district is hilly with many valleys and rivers. There are several villages or parts of villages in Bududa district that are hard-to- reach. The location of Bududa in Uganda and the parishes therein are shown in Figure 1.

### Definition of cholera cases and deaths

The definitions employed in this study are based on Integrated Disease Surveillance and Response (IDSR) guidelines 38 and the national standard case definition for cholera below 39. Four definitions were employed for cholera cases and deaths: 1) Community case definition: Any person with lots of watery diarrhea in an area where a cholera outbreak has been declared, 2) Suspected case definition; Any person aged 5 years or more, presenting with dehydration in an area with no ongoing epidemic, 3) Any death from acute watery diarrhea in an area with cholera epidemic or any person age 2 years or more with acute watery diarrhea, 4) a confirmed cholera case: a suspected case from whose stool sample *V. cholerae* serogroup O1 or O139 has been isolated.

### Data variables retrieved and analysed

#### Variables from the landslide reports

Data on date of landslide occurrence, name of affected villages, number of persons dead, number of persons injured, number of affected households, property damaged, number of persons displaced and alive.

#### Variable from the line lists and situational reports

The following information were collected and analysed on cholera cases meeting the standard case definition for cholera above and from the reports: daily number of cholera cases, daily number of deaths, total cases by village of origin, number of males, number of females, the age, village of residence, parish, sub county, presenting symptoms, treatment given, status of the case in terms of admitted, discharged or died.

#### Variable from laboratory reports

Stool samples of suspected cholera cases were tested and report provided to the Ministry of Health and Bududa district health office to guide the management of cases. The following information was extracted: type of stool sample, number of samples collected, samples that tested positive for *V. cholerae*, type of *V. cholerae* serotype identified, antibiotic sensitivity of the *V. cholerae* organisms present in the stool.

#### Variables on WaSH assessments from reports

All homes in villages listed for having cholera cases were assessed to determine status of WaSH using a simple checklist by a team of trained community health workers selected from the affected villages. The following variables were assessed; the number of household occupants, presence of latrine, the type of latrine, level of cleanness of the latrine, source of drinking water, mode of water storage, availability of hand washing facility, presence of soap for hand washing, general level of home hygiene.

#### Variables from outbreak epidemiological and reactive oral cholera vaccination campaign reports

The following information was extracted from the two reports: date the outbreak was notified to the MoH, date the epidemic was confirmed, date the outbreak was declared, date the MoH decided to use OCV, source of OCV used, date the campaign started, date the campaign ended, target population, strategy used to administer OCV, type of OCV, doses of OCV supplied and doses of OCV administered by administrative area.

### Data management

Data were collected into spreadsheet, cleaned to remove errors and analysed to generate frequencies, percentages, trends and proportions. The data were presented in tables, graphs and maps. The maps were created from shapefiles obtained from the United Nations High Commission for Refugees (UNHCR) 40 by using ArcGIS Version 10.5 41.

### Ethical clearance

This study was conducted as a component of routine integrated surveillance and response to cholera outbreak in Bududa district by the MoH. These types of studies are Institutional Review Board (IRB) clearance exempted. However, clearance to carry out this study was sought from the Uganda Health Research Organization, Ref: UNHRO/res/floods/Bwire/1.08.2022. The data shared were according to that permitted for routine MoH epidemiological surveillance to share with the public. Information shared were anonymous or aggregated and without personal identifiers.

### Role of the funding source

The authors assume full responsibility for the analyses made and interpretation of the data and decision to publish the study findings.

## Results

### Description of the landslides

Data showed that landslides were triggered by the heavy and continuous torrential rainfall that occurred on 4^th^ and 5^th^ June 2019 affecting several villages in Bududa district. There were numerous small landslides that occurred in different parishes of Bududa district but the major ones affected sub-counties of Buwali, Bukalasi, Bumayoka and Bubiita. There were four big landslides that occurred during the two days of heavy rainfall. Two of the landslides were in Buwali sub-county while one was in Bukalasi and another Bumayoka sub-county. These landslides resulted in the deaths of six (6) persons and severe injuries to 27 persons who were retrieved and referred to Bududa general hospital for review, admission and treatment. Overall, 100 households with 669 persons were affected. Due to damage to property, 80 of the affected households (480 community members) were displaced and resettled in the neighbouring villages by friends, relatives and Bududa district Local Government. During the same period (4 - 5^th^ June 2019) as a consequence of the heavy rains, there were floods that burst River *Bugibuni* which is one of the tributaries of River *Manafwa*. These floods destroyed property that included the houses, latrines, crops, water sources and others. Approximately, 111 households with a total of 426 persons were affected. There were no direct flood related deaths (drowning) reported as a result of flood during that week or later.

### Cholera outbreak detection, confirmation and outbreak declaration in Bududa district, June 2019

According to the medical records at the district, the first suspected cholera case was seen on 19^th^ June 2019. This was two weeks from the time the floods occurred. This index case was a nine-year-old female who presented at Bushika Health Centre III with severe watery diarrhea and dehydration. The health workers on duty examined the patient and found that she met the standard case definition for the suspected case of cholera. The health workers rehydrated the case and transferred her to Bushigayi HC III for further management. The staff at Bushigayi HC-III agreed with the suspected cholera diagnosis and transferred the patient into an isolation facility /cholera treatment centre and administered appropriate cholera case management. On 20^th^ June 2019, more suspected cholera cases were seen at Bushika Health Centre III originating from the same village. Fresh stool samples were taken from these suspected cases and shipped to Mbale regional referral hospital laboratory where cholera testing and confirmation of the outbreak was done.

**Figure 2:**
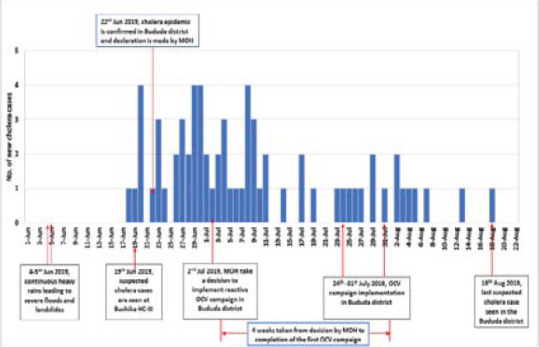
The daily number of reported new cholera cases and the actions taken by the MoH and Bududa district Local Government to control the cholera outbreak

In all, a total of four (4) fresh stool samples were collected and tested of which three (3) samples were confirmed positive on 22^nd^ June 2019 for *V. cholerae*, serotype Inaba. Laboratory test showed that the isolated microorganisms from the stool samples were sensitive to several common antibiotics. The district then communicated to the MoH seeking support to control the outbreak. On 22^nd^ June 2019, the MoH received a report of confirmation of cholera outbreak in Bududa district. The Ministry of Health team reviewed the reports and made consultations after which declaration of a cholera outbreak in Bududa district was pronounced. By the end of the outbreak, the total number of cases and deaths were 67 and one (1) death respectively with case fatality rate of 1.5%. Propagation of the cholera outbreak in Bududa district overtime is shown in Figure 2.

### Laboratory tests results

Fresh stool samples were collected from a cluster of the initial suspected cholera cases and tested to confirm the outbreak. Subsequently, stool samples were taken off from few cases regularly to monitor the outbreak and microbial characteristics. Cumulatively, there were 67 cases reported of these 15 cases had fresh stool samples collected and tested. The test positivity rate was high among the tested stool samples with 14 of these (93%) yielding V.cholerae 01, Inaba serotype. The isolated V. cholerae bacteria showed antibiotic sensitivity to chloramphenicol, cotrimoxazole, ciprofloxacin and tetracycline. However, the organisms were resistant to nalidixic acid.

### Age and sex distribution of the cholera affected cases

**Figure 3:**
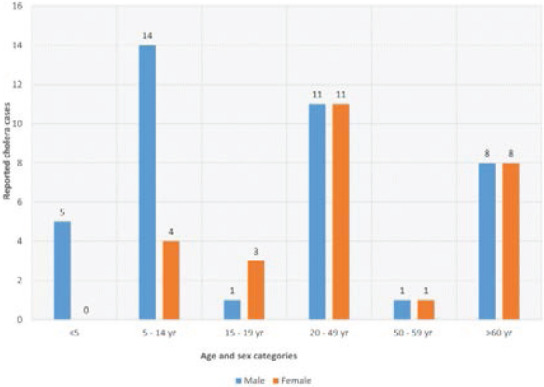
The age and sex distribution of the reported suspected and confirmed cholera cases in Bududa district, 2019

All age groups and sexes were affected. However, in age group 6 – 14 years, the number of males affected were more than their female counterparts. In this age group, the males were more than three times affected by cholera than the females. Age and sex distribution of cases is shown in Figure 3.

### Spatial distribution of cholera cases in Bududa district, 2019

**Figure 4:**
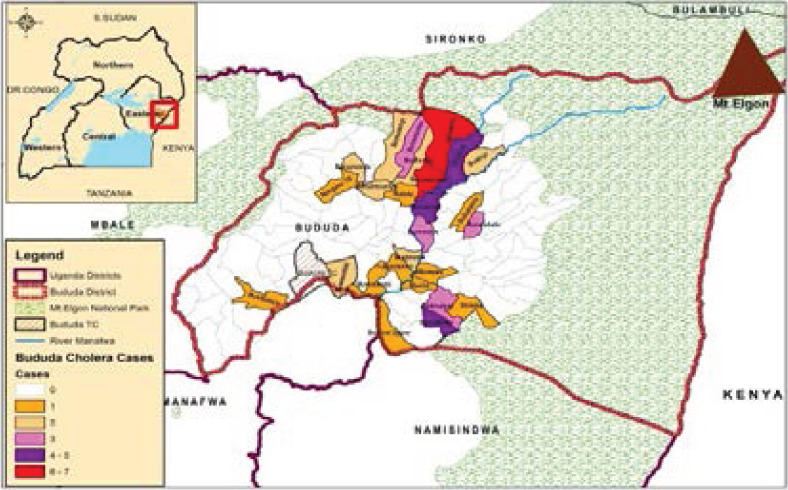
Spatial distribution of the reported cholera cases in Bududa district, 2019

Most of the cholera cases were from the villages and parishes that were affected by landslides and floods. Some of these parishes were located along the tributaries of River Manafwa as shown in Figure 4.

### Water, sanitation and hygiene (WaSH) condition assessment

**Figure 5:**
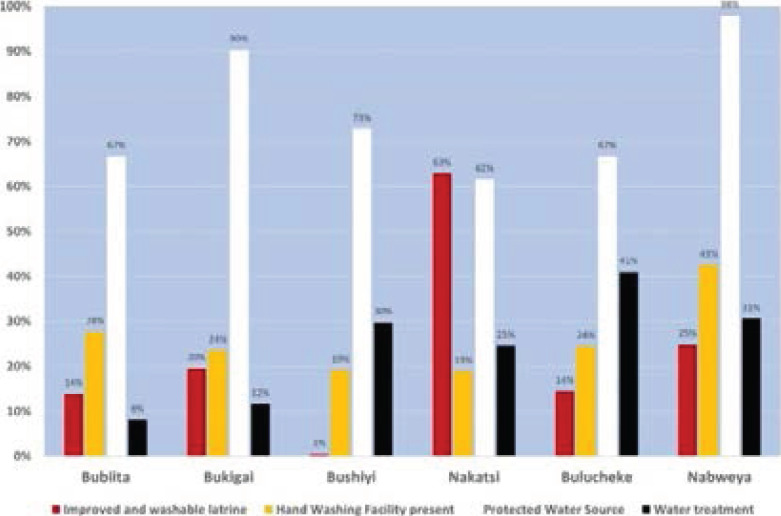
Water, sanitation, hygiene and hand washing coverage of the cholera affected sub-counties in Bududa district, 2019

During the OCV campaigns, a total of 4,727 households were visited and assessed to establish status of WaSH in households. Majority of households 76% (3,600/4,727) used water from protected sources, however 34.2% (1,617/4,727) did not apply any treatment method of household water treatment. In regards to the availability of sanitary facilities, 19.7% (931/4,727) did not have a latrine facility. Only 24.2 % (879/3,628) had an improved latrine that had washable floors, 26.8% (1,023/3,818) had hand washing facilities with soap and WaSH coverage in the affected sub-counties were as shown in Figure 5.

### Oral cholera vaccination campaign coverage

Oral cholera vaccine mass campaign targeted 52,023 persons in the parishes that reported cholera cases. The administrative OCV coverage for both the first and second OCV doses were high and, in some parishes, above 100% since people in the neighbourhood were also interested. OCV coverage by parish are shown in Table 1.

**Table 1:** Oral cholera vaccine coverage by the affected parishes in Bududa district, 2019

Parishes Vaccinated	Target	Persons Vaccinated OCV Round 1	Round 1 coverage (%)	Persons Vaccinated OCV Round 2	Round 2 Coverage (%)	Persons Received 1st dose during R2	Total vaccinated in Round 2
Maaba	1,342	2,067	154	1,338	99	215	1,553
Shihulusi	1,480	1,633	110	1,470	99	175	1,645
Buwashi	1,202	1,520	127	1,198	99	165	1,363
Bumakhwa	715	1,724	241	708	99	123	831
Bumusi	996	1,472	148	986	98	148	1,134
Bumatanda	2,046	2,296	112	1,698	83	420	2,118
Bunaporo	2,157	2,546	118	1,812	84	424	2,236
Bukibokolo	4,771	5,363	112	3,951	83	345	4,296
Busiliwa	2,578	3,009	117	2,580	100	345	2,925
Bushiyi	3,002	2,633	88	2,957	98.5	123	3,080
Bubukasha	1,627	2,760	170	1,641	100	154	1,795
Bunambatsu	3,107	2,788	90	2,665	86	65	2,730
Bushiwunya	3,059	2,916	95	2,695	88	33	2,728
Bumusenyi	3,856	3,455	90	3,363	87	43	3,406
Bunamanda	2,124	2,885	136	1,812	85	22	1,834
Bumwalukani	3,514	3,120	89	3,117	88	458	3,575
Saskusaku	4,772	3,663	77	3,657	76	593	4,250
Bumwalye	2,124	2,861	135	2,758	129	160	2,918
Bumasata	1,472	1,902	129	1,788	121	118	1,906
Bunandutu	2,050	5,241	256	2,037	99.3	214	2,251
Bunatsmya	1,509	2,438	162	1,492	98.8	149	1,641
Bulobi	2,521	2,784	110	2,500	99.1	284	2,784
**22**	**52,023**	**6,1076**	**117**	**48,223**	**92.3**	**4,776**	**52,999**

## Discussion

This study showed that in early June 2019, Bududa district experienced catastrophic landslides and flash floods that were later followed by a cholera epidemic. There were multiple major public health emergencies affecting the same population and communities in the district over a short time frame. Fortunately, the cholera epidemic was detected early and rapidly controlled by implementation of a package of targeted measures that included among others reactive oral cholera mass vaccination campaign. The response to control the cholera epidemic was unique in several ways. First, in contrast to the previous reactive OCV mass vaccination campaigns in Uganda and elsewhere where the source of OCV was GTFCC, Geneva, Switzerland 17,42, in this case the OCV stockpile/ reserve was within Uganda and not requiring shipment. Second, there was little or no need for the paper work and complex application processes and approvals for the vaccines to be deployed to the affected community since authorization to use the vaccines was in the country by the MoH top leadership with support from the WHO- country office. Third, the time taken from decision to use of OCV by MoH for the reactive campaign on 22^nd^ June 2019, to completion of the first campaign on 8^th^ July 2019, was four (4) weeks. This was shorter time compared to similar reactive mass OCV campaign to control cholera epidemic during major public health emergencies such as was the case of the 2018 cholera epidemic in Hoima district, in mid-western Uganda where the campaign was conducted after the outbreak had subsided 17. In this study, the time for OCV intervention was also shorter than that used during reactive OCV campaigns in other countries in Africa such as South Sudan and Nigeria 16,18. Fourth, the beneficiary of OCV campaign were extremely targeted to those specific parishes with the highest risk of infection or where cholera cases had been reported as opposed to the entire sub-county (5 or more parishes). Consequently, the cholera epidemic was rapidly controlled. The rapid epidemic control had potential to prevent cholera spread to other districts in Eastern region that were experiencing heavy rainfall and floods during the same time.

Most importantly, though Bududa district is a hard-to-reach area due to the challenging terrains, floods and land-slides effects, the time interval from decision to use OCV for epidemic control to implementation of OCV campaign was very short. Also, the OCV coverage for both first and the second doses were high which increased the impact of the OCV campaign on the cholera epidemic. In some parishes the OCV coverage were above 100% possibly due to the high acceptability of the OCV by the affected communities and the influx of people from the surrounding parishes and sub-counties which had not been targeted. Furthermore, the affected communities appreciated the impact of cholera on their health and cooperated with the health workers. Other possible reason for the high coverage could be due to strategy used where the health workers moved from home to home which allowed for all homes to be reached.

Despite the destruction of infrastructure and disruption of social services caused by landslides and floods, only one death was reported from the 2019 cholera outbreak in Bududa District. This could be due to resettlement of affected people in camps away from hard-to-reach areas, the presence of emergency responders and rapid mass OCV campaigns. On the other hand, the WaSH conditions coverage was found to be low and without vaccination, this scenario could provide suitable conditions for transmission of cholera as was observed previously in Tanzania 43 and in South Africa 44.

Cholera kills and spreads rapidly 45 and the incubation period ranges from a few hours to five days 46 yet the current requisition procedure for OCV, approval of the request, shipment of vaccines, to actual implementation of the campaign takes 2 – 3 months or more 16–18. However, when the vaccines are prepositioned in endemic setting as was the case with the vaccine stockpile used in Bududa reactive mass vaccination campaign, it is possible for the duration to be less than one month or days to one (1) week for an emergency medicine delivery order from the Uganda National Medical Stores. Furthermore, by decentralizing / prepositioning OCV in endemic countries it is possible that the cost of implementation of the OCV campaign could be reduced by integration of OCV into routine vaccination schedules in cholera affected districts. The findings of this study address the knowledge and practice gap regarding feasibility of stockpiling/prepositioning of OCV in cholera endemic countries verses the status quo where OCV is located somewhere in Europe or Asia awaiting finalization of lengthy approval procedures and shipment. Currently, there is no local production of OCV on the African continent although under the auspices of the new public health order of the Africa Centres for Disease Control and Prevention (Africa CDC), there are efforts to explore local vaccine manufacturing on the African continent. We think that these promising steps of having local OCV stockpile in affected Partner States and OCV production on African continent should be first tracked to enable the countries in sub-Saharan Africa eliminate cholera by 2030 as per WHO cholera roadmap 14.

As part of the process to strengthen capacity for epidemic preparedness and response (EPR), prior knowledge of risk and vulnerability profile of an area are very important. Taking for example risk factors, such as water sources contaminated with faecal material as was the case in Bududa district following heavy rains and landslides, the potential for cholera to spread and affect many people in short time, it is very important to quickly institute a package of known prevention and control interventions 47. This is critical and calls for review of guidelines regarding rapid access and timely implementation of reactive OCV campaigns to enhance effectiveness of the interventions for prevention and control of cholera. Therefore, quick decision making that goes with rapid implementation of mass OCV campaign has potential to curtail the spread of cholera and attain quick control of the outbreak.

Cholera is one of the major health issues affecting several countries each year 48. Oral cholera vaccine stockpile for reactive campaigns is accessed through application to International Coordinating Group (ICG). However, some countries experiencing cholera outbreaks have not accessed these vaccines 49. There could be several reasons for this relatively low access to OCV despite the morbidity and mortality from cholera in the endemic countries being an important health issue 50. One, the requisition process involves to and from communication and often is time consuming. Time to focus on outbreak control is lost in technicalities where by countries have to conduct risk assessment, write report and send it to WHO secretariat in Geneva, Switzerland. Often, there is time constraint on responders who also have to oversee known effective cholera control interventions (case management, surveillance, promotion of access to safe water and sanitation among others) to save lives. Most importantly, the availability of OCV stockpile within Uganda facilitated policy-makers to discuss and decide to implement OCV campaign in shortest time possible since powers of advisory and decision on vaccination were vested in them.

In this study, the area targeted were the parishes (5,000 persons) as opposed to sub counties (20,000 - 50,000 persons) in Hoima district OCV campaign 17. Since access to OCV is still limited and not affordable by most cholera endemic countries, studies are needed to guide stakeholders on ideal administrative unit to target for mass OCV vaccination. Furthermore, given the resurgence of cholera in African continent 50, continental and regional actors/bodies (Africa Centres for Disease Control (Africa CDC), East African Community (EAC), Economic Community of West Africa (ECOWAS), Southern African Developmental Community (SADC) and Intergovernmental Authority on Development (IGAD)) which are closer to the affected countries/communities should consider stockpiling OCV and empower national authorities to supplement the GTFCC efforts.

### Strength and weakness of this study

This study combined cholera control with assessment of WaSH conditions through community survey which generated data to guide disaster recovery process. This information is important for planning and prevention of future cholera outbreaks. Use of the community health workers to conduct assessment increased community knowledge and participation in finding local solution for community problem. It may not be possible to generalize our finding to other States since there may be differences in reporting, detection and declaration of cholera outbreaks.

## Conclusion

This study shows that Bududa district experienced heavy rains with floods and catastrophic landslides that were followed by cholera outbreak. However, early detection and institution of control measures that included targeted mass OCV campaign using OCV stockpile that was in Uganda at the time of the disaster, facilitated quick field deployment of OCV and ultimately rapid control of the cholera outbreak. Furthermore, it is possible that this rapid intervention prevented cholera spread to surrounding districts with similar risk factors within the region. Therefore, countries at high risk of cholera outbreaks should stockpile OCV for future emergency rapid deployment. As we strive to attain the Africa Union agenda 2063 “the Africa we want” with capacities and capabilities to detect and respond to public health threats. We recommend Africa CDC to fast track the local manufacturing of essential commodities such as OCV to meet the growing demand on the continent.
